# DDB2 regulates Epithelial-to-Mesenchymal Transition (EMT) in Oral/Head and Neck Squamous Cell Carcinoma

**DOI:** 10.18632/oncotarget.26168

**Published:** 2018-10-05

**Authors:** Prashant V. Bommi, Sriram Ravindran, Pradip Raychaudhuri, Srilata Bagchi

**Affiliations:** ^1^ Department of Oral Biology, College of Dentistry, University of Illinois at Chicago, Chicago, Illinois, USA; ^2^ Department of Biochemistry and Molecular Genetics, College of Medicine, University of Illinois at Chicago, Chicago, Illinois, USA; ^3^ Current Address: Department of Clinical Cancer Prevention, Biological Sciences Research Building (BSRB), University of Texas MD Anderson Cancer Center, Houston, Texas

**Keywords:** oral/head and neck squamous cell carcinoma (HNSCC), damaged DNA binding protein 2 (DDB2), epithelial-to-mesenchymal transition (EMT), transforming growth factor beta (TGFβ), tumor suppressor

## Abstract

DDB2 is a sensor of DNA damage and it plays an important role in Global Genomic Repair (GG-NER). Our previous studies show that DDB2 is involved in the regulation of metastasis in colon adenocarcinoma. Squamous Cell Carcinomas in the Oral/Head & Neck region (HNSCC) are particularly aggressive due to high incidence of recurrence and distant metastasis. In this study, we show that DDB2 expression is downregulated in advanced HNSCCs and loss of DDB2 expression coincides with reduced survival. Recent meta-analysis of gene expression data characterized the mesenchymal-type (EMT-type) as one most aggressive cancer cluster in HNSCC. Here, we report that DDB2 constitutively represses mRNA expression of the EMT- regulatory transcription factors *SNAIL*, *ZEB1*, and angiogenic factor VEGF in HNSCC cells. As a result, re-expression of DDB2 in metastatic cells reversed EMT with transcriptional upregulation of epithelial marker E-cadherin, and downregulation of mesenchymal markers N-cadherin, Vimentin, and Fibronectin. Interestingly, in a reverse assay, depletion of DDB2 in non-metastatic cells induced expression of the same EMT-regulatory transcription factors. TGFβs are major regulators of Snail and Zeb1, and we observed that DDB2 transcriptionally regulates expression of *TGFB2* in HNSCC cells. Re-expression of DDB2 in mouse embryonic fibroblasts (MEFs) isolated from Ddb2 (−/−) knockout-mice resulted in repression of EMT-regulatory factors Zeb1, Snail and *Tgfb2*. Taken together, these results support the active role of DDB2 as a candidate suppressor of the EMT-process in HNSCC. Early detection leads to significantly higher survival in HNSCC and DDB2 expression in tumors can be a predictor of EMT progression.

## INTRODUCTION

Squamous Cell Carcinomas in the Oral/Head & Neck region (HNSCC) are particularly aggressive due to high incidence of recurrence and distant metastasis. HNSCC is the eighth most common malignancy and an estimated 600,000 new cases are diagnosed worldwide each year, with more than 350,000 deaths annually [1, 2]. The vast majority (>90%) of HNSCCs are epithelial in origin and produce squamous cell carcinoma [3]. Late Stage III/IV tumors are large and show massive invasion of nearby or distal structures (reviewed in [4]. Early detection leads to significantly higher survival; there is profound need for understanding the mechanisms of progression towards aggressive lesions and development of useful prognostic biomarkers in HNSCC [5].

A recent meta-analysis of gene expression data from HNSCC characterized six different clusters. One most aggressive cancer cluster belong to a mesenchymal-type (EMT-type) [6]. Epithelial to Mesenchymal transition (EMT) is an evolutionarily conserved developmental process, during which, the epithelial cells undergo molecular changes that lead to a conversion of the epithelial phenotype towards an mesenchymal phenotype [7]. EMT is marked by loss of epithelial markers including E-cadherin, β-catenin, and gain of mesenchymal markers such as Vimentin, Fibronectin, and Smooth muscle actin. EMT is often exhibited during metastatic progression of tumors of epithelial origin [8]. Several oncogenic pathways induce EMT including activated Src, H-Ras, ETS, Notch, and NF-kB. Among different signaling pathways, TGFβ, Wnt/b-catenin, and hypoxia have profound role on EMT-progression [9–11]. Altogether, these diverse pathways induce EMT through activation of a set of transcription factors (EMT-TFs) including Snail, Slug, Zeb1, Zeb2, and Twist [12]. TGFb1 and Wnt/b-catenin mediated EMT induction involves activation of Snail, a known repressor of E-cadherin [11–13]. The TGFb effectors Smads associate with ZEB proteins to repress expression of E-cadherin [10]. Hypoxia induced transcription factor HIF1α induces EMT by activating expression of *ZEB1*, *TWIST*, *SNAIL*, and *VEGF* [9, 14–16]. In this study, we show that DDB2 blocks EMT in mesenchymal HNSCC cells and revert the process towards epithelial transition (MET).

DDB2 is encoded by the nucleotide excision repair (NER) gene, Xeroderma Pigmentosum complementation group E (XPE) [17–19]. XPE is a rare autosomal recessive genetic disorder characterized by defective DNA repair with markedly increased risk of developing skin cancer associated with exposure to environmental carcinogens and UV [20, 21]. Several reports have described that DDB2 is required for the recognition and removal of DNA lesion presented by UV-light induced cyclobutane pyrimidine dimers (CPD) and 6–4 pyrimidine-pyrimidone dimers (6–4 PPs) [22–24]. Besides its role in NER, DDB2 along with its heterodimer DDB1 associates with Cullin4 to form an E3 ubiquitin ligase complex. In this complex, DDB1 attaches to Cul4A/B and acts as the linker protein, while DDB2 binds to DDB1 and functions as substrate receptor molecule. Cul4-DDB1-DDB2 ub-ligase targets DNA repair protein XPC, and cell cycle regulators p21 and p27 to ubiquitin-mediated proteolysis [25–27].

Recent studies by others and us demonstrate that DDB2 is involved in transcriptional regulation of tumor promoting oncogenes, as well as, tumor suppressor genes. The DDB2 knockout (DDB2-KO) MEF's show deficiency in accumulation of reactive oxygen species (ROS) [26]. An independent study show that DDB2 transcriptionally represses the anti-oxidant gene MnSOD in breast cancer [28]. Recent studies from our lab revealed that DDB2 is a potent regulator of EMT and metastasis in colon adenocarcinoma cells, the mechanism indicated the transcription regulatory function of DDB2 in repressing expression of pro-EMT transcription factors (EMT-Tfs), *SNAIL* and *ZEB1* [29]. Roy *et al*., showed that DDB2 recruits histone methyl transferase, Suv39h, at the promoters of *SNAIL* and *ZEB1* genes and altered the levels of H3K9Me3, H3K14Ac on the promoter [29]. In a separate study, DDB2 was found to repress the anti-apoptotic gene, Bcl-2, in human ovarian cancer cells [30]. The underlying mechanism revealed the recruitment of HDAC1 by DDB1 to deacetylate H3K9 and H3K14 across the regulatory regions of the Bcl-2 promoter [30]. Over-expression of DDB2 was found to inhibit the self-renewal property and tumorigenecity of ovarian CSCs by suppressing the NF-κB pathway and stem cell marker, *NANOG* [31]. Thus, DDB2 functions converge on inhibiting cancer promoting events and DDB2 itself is found to be directly involved in lesion-independent binding to DNA and suppressing the transcription of genes directly involved in tumor progression. In this study, we describe the significance of loss of DDB2 expression in HNSCC and a role of DDB2 as a dominant repressor of EMT.

## RESULTS

### Reduced expression of DDB2 coincides with aggressive progression of HNSCC

**Figure 1:**
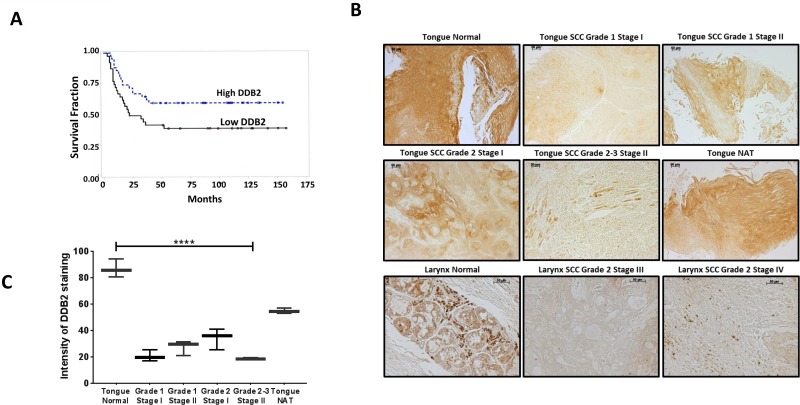
Lower DDB2 expression in HNSCC tumor tissues (**A**) A representative Kaplan–Meier analysis of overall survival of HNSCC patients (*n* = 81) stratified according to DDB2 expression in tumors. Patients with higher DDB2 expression survived longer compared to patients with lower DDB2 expression, significant at alpha level of 0.05 (log rank *p* = 0.0404). (**B**) Loss of DDB2 expression in HNSCCs. DDB2-immunohistochemistry of human tissue microarrays (US Biomax # HN242a and HN811a). Representative images from normal tongue tissue, tongue SCCs of grades 1–3 stage I–III and cancer adjacent normal tongue tissue (NAT), normal larynx tissues and larynx SCCs grade 2 stage III and IV are shown. Scale bar for all the images, 10 μm. (**C**) The average intensity of DDB2 staining of normal tongue versus tongue SCCs showed lower staining in all SCC tissues and acute loss of staining in advanced SCCs. *p* < 0.0001.

Metastatic progression of cancer often coincides with reduced expression of DDB2 [32]. For example, DDB2 is down regulated in metastatic breast [33] and colon cancer [29]. Kaplan–Meier analysis of overall survival in HNSCC patients (*n* = 81) from the publicly available (Oncomine) dataset showed reduced mRNA expression of DDB2 (low DDB2 *n* = 39) predicts poor prognosis compared with high DDB2 expression (*n* = 42) with significance at *p* = 0.0404 (Figure 1A). We previously reported loss of DDB2 expression in high-grade colon tumors of both human and mice origin [29]. In this study, we analyzed expression of DDB2 protein in HNSCC tumor tissue microarrays; (1) US-Biomax # HN811a containing 19 cases of tumors, six adjacent normal tissues (NAT), two normal tissues (tongue), triplicate cores per case, and (2) US-Biomax # HN242a containing 9 cases of HNSCCs, and two normal tissues of tongue and larynx in duplicates. In both arrays, we observed significantly lower expression (>1.5 fold) of DDB2 at all stages of tongue SCCs and larynx SCCs (Figure 1B, 1C). In contrast, we observed significantly higher expression of DDB2 in both normal tongue tissues and cancer adjacent normal tissues (NAT). A representative DDB2 expression data in normal tongue tissues, tongue SCCs, normal larynx and larynx SCCs from US-Biomax arrays is shown in Figure 1B. Relative expression of DDB2 in normal tongue and tongue SCCs is presented in Figure 1C. These results clearly establish reduced expression of DDB2 protein in aggressive HNSCC tumors in comparison to normal tissue.

### Lower DDB2 expression in HNSCC cell lines

**Figure 2:**
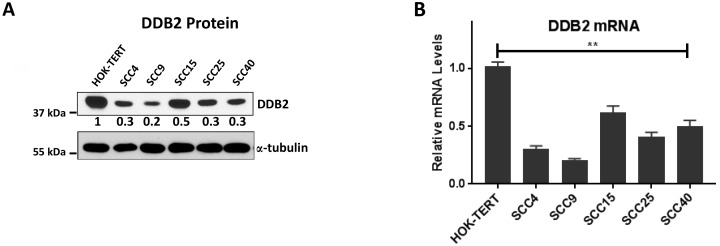
Lower DDB2 expression is HNSCC cells (**A**) Expression of DDB2 at the protein level was determined by western blot analysis of total cell lysates (30 ug) of HOK-TERT (TERT-immortalized human oral keratinocytes) and HNSCC cell lines, SCC4, SCC9, SCC15, SCC25, and SCC40. a-tubulin was used as a loading control. Relative expression of DDB2 in SCC-cell lines were normalized against HOK-TERT, quantitation was conducted by densitometric analysis using ImageJ software (**B**) The relative mRNA expression of DDB2 in HOK-TERT, SCC4, SCC9, SCC15, SCC25, and SCC40 cells were analyzed by SYBR green based qRT-PCR. Data was normalized to the mRNA levels of Cyclophilin and expressed as fold changes against the HOK-TERT cells. Mean of three independent amplifications performed in duplicates, error bars are shown.

Next, we analyzed the expression of DDB2 in five well-characterized HNSCC cell lines, SCC4, SCC9, SCC15, SCC25, and SCC40 and compared it to the expression in TERT-immortalized normal human oral keratinocytes (HOK-TERT). In comparison to HOK-TERT cells, we observed significantly lower expression of DDB2 protein in four out of five cell lines and significant downregulation of DDB2 mRNA in three cell lines, SCC4, SCC9 and SCC25 (Figure 2A, 2B). Interestingly, we observed that a less aggressive cell line SCC15 expresses both DDB2 mRNA and protein at higher level compared to cell lines derived from aggressive HNSCCs such as SCC4 and SCC9 [34]. Chromosomal mutations in DDB2 cDNA in XPE patients are associated with repair deficiency and increased tumor susceptibility [20, 22, 35]. We analyzed DDB2 transcripts by RT-PCR and sequencing in two cell lines SCC9 and SCC4 that showed acute repression of DDB2 mRNA and protein, but none of them harbors any mutation (data not shown). Taken together, these results show that expression of DDB2 in aggressive HNSCC cell lines are significantly lower at both protein and RNA levels (Figure 2A, 2B).

### Expression of DDB2 in SCC9 cells induces mesenchymal to epithelial-like (MET) changes

**Figure 3:**
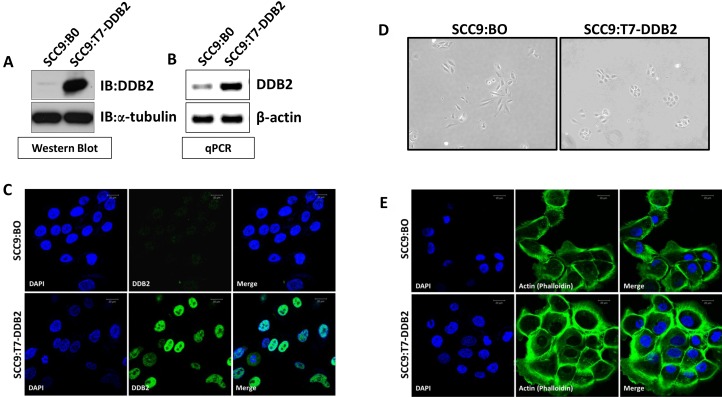
DDB2 reverses mesenchymal phenotype Stable pool of SCC9 cells were generated by transduction of either control retrovirus Babe-puro (SCC9:BO) or T7-DDB2 expressing retrovirus (SCC9:T7-DDB2). Expression of DDB2 was analyzed by (**A**) western blots of cell lysates (20ug) using a-tubulin as loading contrl, (**B**) DDB2 mRNA level were analyzed by qRT-PCR using cyclophilin as loading control, and (**C**) DDB2 localization was analyzed by immunocytochemical analysis with DDB2-Ab and counterstained with DAPI. (**D**) A representative phase contrast image (10×) of SCC9:B0 and SCC9:T7-DDB2 is shown. (**E**) SCC9:BO and SCC9:T7-DDB2 cells were subjected to immunocytochemical analysis using fluorophore, FITC-conjugated Phalloidin and counterstained with DAPI.

The survival rates of non-HPV HNSCCs depend largely on progression to metastasis [1]. Previous report from our lab showed that DDB2 acts as a barrier to EMT/metastasis in colon cancer cells [29]. We also showed that reduced expression of DDB2 in colon cancer cells leads to increased migration/invasion and tumorigenesis [29]. To determine the role of DDB2 in regulating EMT in HNSCC, we chose two cell lines, the non-mesenchymal SCC15 expressing higher level of DDB2 mRNA and protein and mesenchymal SCC9 expressing significantly lower level of DDB2 mRNA and protein (Figure 2). We made a polyclonal stable cell line (SCC9:T7-DDB2) expressing T7-tagged DDB2 in SCC9. Overexpression of DDB2 in these cells were confirmed at the protein level using western blot assay, and at the transcript level using qRT-PCR analysis (Figure 3A, 3B). Immuno-cytochemistry showed expected nuclear localization (Figure 3C). The parental SCC9 expressing control plasmid, SCC9:BO cells exhibit elongated mesenchymal-like morphology, and we found that SCC9:T7-DDB2 cells exhibit more cuboidal like morphological features similar to epithelial phenotype (Figure 3D). Actin (Phalloidin) staining showed SCC9:T7-DDB2 cells had a tighter cytoskeletal network in comparison to control SCC9:BO cells (Figure 3E).

**Figure 4:**
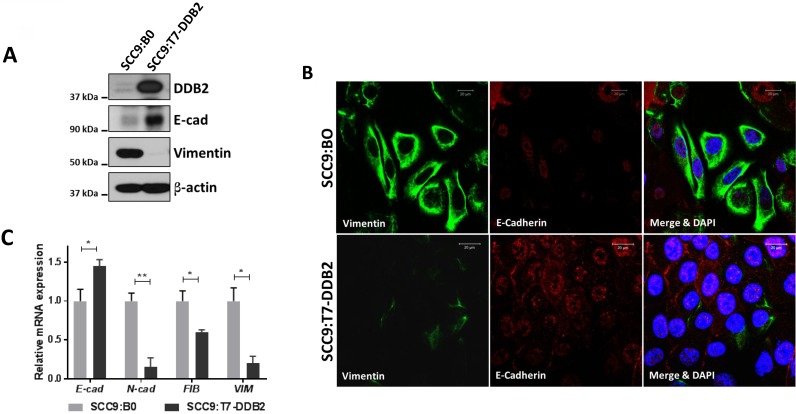
DDB2 inhibits EMT in SCC9 cells and reverses mesenchymal phenotype (**A**) Immunoblots of SCC9:B0 or SCC9:T7-DDB2 cell lysates showing expression of DDB2, E-cadherin, and Vimentin. Total cell lysates (20 ug) were probed with the indicated antibodies using western blot assay. β-actin was used as a loading control. (**B**) SCC9:B0 or SCC9:T7-DDB2 cells were co-stained with Vimentin (Green) and E-cadherin (Red) and imaged with confocal microscopy. The cell nuclei were counterstained with DAPI (Blue). Representative confocal images are presented. Scale bar = 20 μM. (**C**) Relative mRNA expression of EMT-markers, E-cadherin, N-cadherin, Fibronectin and Vimentin in SCC9:BO and SCC9:T7-DDB2 cells are shown. Columns, mean of three independent amplifications performed in duplicate; error bars, St. Dev. ^*^P < 0.05, ^**^P < 0.01 (Student's unpaired *t* test).

Loss of epithelial marker E-cadherin and gain of mesenchymal makers Vimentin and N-cadherin are hallmarks of EMT and we assayed for these markers in SCC9:BO and SCC9:T7-DDB2 cells. Using western blot assays, we observed significant loss of Vimentin and N-cadherin and increased expression of E-cadherin in SCC9:T7-DDB2 cells (Figure 4A). Immunocytochemical analysis verified the western blot result; we observed a dramatic reduction in Vimentin staining and a modest increase in E-cadherin staining in SCC9:T7-DDB2 cells (Figure 4B). The mRNA expression analysis using qRT-PCR further confirmed reduced expression of three mesenchymal markers Vimentin, N-cadherin and Fibronectin mRNA in SCC9:T7-DDB2 cells (Figure 4C). These results are consistent with the role of DDB2 in reversing EMT in SCC9 cells. This reversible phenotypic change is termed as mesenchymal to epithelial transition (MET) and these observations show that re-expression of DDB2 restored epithelial phenotype in metastatic SCC9 cells. This MET-like change would be consistent with a metastasis suppression function of DDB2 in HNSCC.

### DDB2 represses EMT-regulatory factors in HNSCC

**Figure 5:**
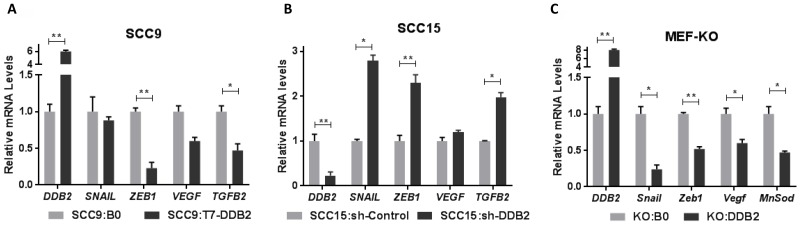
DDB2 represses expression of EMT-regulatory genes in HNSCC and in DDB2 knock-out MEFs (mouse embryonic fibroblasts) Total RNA isolated from (**A**) SCC9:B0 and SCC9:T7-DDB2 cells, (**B**) SCC15:sh-Control and SCC15:sh-DDB2 cells were subjected to qRT-PCR to analyze the mRNA expression of DDB2, EMT-regulatory transcription factors SNAIL and ZEB1, angiogenic actor *VEGF*, and EMT-regulatory cytokine *TGFB2*. (**C**) The mouse embryonic fibroblasts (MEF-KO) isolated from Ddb2 knock-out (DDB2−/−) mice were transduced with retroviruses containing either control vector (KO:BO) or DDB2 cDNA (KO:DDB2). Total RNA isolated from KO:BO and KO:DDB2 cells were subjected to qRT-PCR analysis for DDB2 and mouse EMT-regulatory genes *Snail*, *Zeb1*, *Vegf* and *MnSod*. Columns, mean of three independent amplifications performed in duplicate; error bars, St. Dev. ^*^P < 0.05, ^**^P < 0.01 (Student's unpaired *t* test).

EMT is stimulated by a myriad of environmental signals including reduced oxygen, cytokines and growth factors that converge to induce a limited number of transcriptional repressors (EMT-TFs) including Snail, Slug, Twist, Zeb1 and Zeb2 [12, 36, 37]. We observed that ectopic expression of DDB2 in SCC9:T7-DDB2 cells led to transcriptional repression of two major EMT-TFs *SNAIL* and *ZEB1*, the angiogenic factor *VEGF* and the EMT-regulatory cytokine TGFB2 (Figure 5A). In a reverse experiment; we made stable pool of SCC15 cells expressing control-shRNA (SCC15:sh-Control) and DDB2-shRNA (SCC15:sh-DDB2). Interestingly, inhibition of DDB2 expression in SCC15:sh-DDB2 cells led to transcriptional activation of *SNAIL*, *ZEB1*, *VEGF* and *TGFB2* (Figure 5B).

To confirm the role of DDB2 in regulation of the key EMT-regulatory genes in a genetic background, we assayed these genes using mouse embryonic fibroblasts (MEFs) isolated from Ddb2 knock-out (KO) mice. The Ddb2 knock out mice are tumor prone [38] and in response to UV-damage or on exposure to mutagens produce skin cancer and colon adenocarcinomas [39, 40]. Comparison of gene expression in WT-MEFs isolated from Wild-type mice and KO-MEFs isolated from DDB2 knock-out mice, revealed higher expression of Snail, Zeb1 and *Tgfb2* in KO-MEFs (data not shown). To explore direct DDB2-regulation of these genes, we restored DDB2 expression in immortalized MEF-KO cell line to generate KO-DDB2 cell line (Figure 5C). Comparison of mRNA expression in KO:BO (control) and KO:DDB2 cells show repression of the same EMT regulatory genes including Snail, Zeb1, *Vegf* and *Tgfb2* in KO:DDB2 cells (Figure 5C). A previous study showed that DDB2 constitutively represses the antioxidant Manganese Superoxide Dismutase (MnSOD) gene by binding through a specific promoter sequence in breast cancer cells (28). In this study, we observed less expression of *MnSod* mRNA in KO:DDB2 cells, which confirms that the transcription regulatory function of DDB2 observed in cancer cells is conserved in MEFs (Figure 5C). These observations strongly support the notion that DDB2 is a critical repressor of EMT-regulatory factors irrespective of cell type or tumor type.

### DDB2 is a major regulator of *TGFB2* gene expression

**Figure 6:**
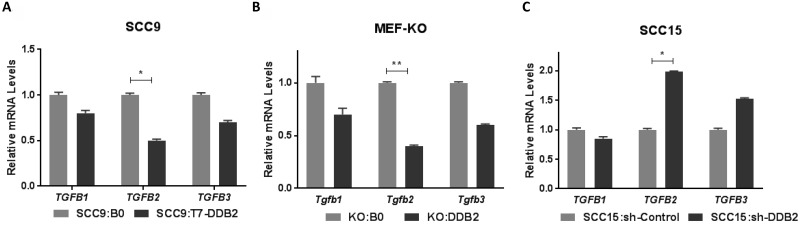
DDB2 is a regulator of *TGFB2* gene expression Total RNA from (**A**) SCC9:B0 and SCC9:T7-DDB2 cells, (**B**) Control MEFs KO:BO and DDB2 expressing KO:DDB2 cells and (**C**) SCC15:sh-control and SCC15:shDDB2 cells were subjected to qRT-PCR analysis. Human TGFβ isoforms, *TGFB1*, *TGFB2*, *TGFB3* and mouse specific Tgfb isoforms *Tgfb1*, *Tgfb2*, *Tgfb3* were analyzed. mRNA expression were normalized against the mRNA level of cyclophilin and expressed as fold changes compared to control cells. Columns, mean of three independent amplifications performed in duplicate; error bars, St. Dev. ^*^P < 0.05, ^**^P < 0.01 (Student's unpaired *t* test).

Among different signaling pathways, the Transforming growth factor βs (TGFβs) have profound effects on EMT progression [10, 13, 41, 42]. Therefore, identification of *TGFB2* gene as a target of DDB2 in HNSCC cells is an important observation. Among the three isoforms of TGFβs, we observed 2-fold decrease in *TGFB2* mRNA in SCC9:T7-DDB2 cells (Figure 6A) and similar increased expression in SCC15:sh-DDB2 cells (Figure 6C). Restoring expression of DDB2 in KO:DDB2 cells led to moderate repression of all Tgfβ isoforms but more than 2-fold repression in *Tgfb2* (Figure 6B). To analyze DDB2 regulation of *TGFB2* in other cancer cells, we compared the expression of the *TGFB2* mRNA in colon adenocarcinoma cell lines HCT116:sh-Control and HCT116:sh-DDB2 cells, and observed a 3-fold induction in HCT116:sh-DDB2 cells (data not shown). Taken together, these results show that DDB2 represses *TGFB2* mRNA expression irrespective of tumor types. Other than EMT-regulation, TGFβs have profound effects on tumor micro-environment, the role of TGFβ2 in DDB2-mediated EMT-regulation in HNSCC tumors needs further investigation.

## DISCUSSION

A comprehensive genomic characterization of HNSCCs shows association with profound genomic instability and alteration in many genes [43]. In this study, we report reduced expression of DDB2 in HNSCC and show that lower DDB2 expression is linked to overall survival of HNSCC patients (Figure 1A). We also observed acute repression of DDB2 protein expression in malignant tongue/larynx SCCs in comparison to normal tissues and cancer adjacent normal tissues (NAT) (Figure 1B). Furthermore, in five well-characterized HNSCC cell lines, we observed decreased expression of both DDB2 protein and mRNA (Figure 2). Interestingly, acute repression of both DDB2 mRNA and protein were seen in two mesenchymal HNSCC cell lines, SCC4 and SCC9 as compared to non-metastatic SCC15 cells.

Overall, DNA repair efficiency plays a key role in progression of HNSCC. For example, polymorphisms in XRCC1 and XPD demonstrated positive association with an increased risk of HNSCC [44]. Polymorphisms in other NER genes including ERCC1, ERCC2, ERCC3, ERCC4, ERCC5, and XPA have also been reported to pose as an increased risk in Laryngeal Cancer [45–47]. Our recent studies demonstrated a DNA damage-independent interaction between DDB1/DDB2 and XRCC5/XRCC6 in colon cancer cells [48]. XPE gene product DDB2 is involved in GG-NER (global genomic nuclear excision repair). XPE patients carry chromosomal mutations in DDB2 gene that result in DNA repair deficiency [49]. We analyzed sequences of DDB2 transcripts isolated from SCC9 and SCC4 cells that express lowest level of DDB2 and found no mutation (data not shown). Mutations in NER genes have not been reported in HNSCC. DDB2 gene is a transcriptional target of the tumor suppressor p53 [50]. p53 mutation is a frequent event in HNSCC [51] and all five HNSCC cell lines used in this study carry p53 mutations and mutations in p19 ARF, a regulator of p53 proteolysis [34, 51]. Therefore, overall lower DDB2 expression in these cell lines might be linked to nonfunctional p53, however differential expression of DDB2 among the cell lines do not correlate with p53 mutation. DDB2 is ubiquitinated by DDB1/Cullin4A-B ubiquitin ligase and is proteolyzed in response to UV-irradiation [25, 52]. Interestingly, Cullin4A gene is amplified in breast cancers [53], and is deregulated in lung cancer [54], information about deregulation of Cul4A/B in HNSCC is currently unknown. Lower DDB2 expression will result in reduced NER activity, which might contribute to evolution of aggressive HNSCCs. However, the mechanism of reduced expression of DDB2 in metastatic HNSCC is currently unknown.

In this study, we show that restoration of DDB2 expression in mesenchymal SCC9 cells lead to reversal of EMT to MET. In a reverse setting, lowering DDB2 expression using shRNA in SCC15:sh-DDB2 cells lead to EMT-like effects. Therefore, DDB2 expression dictates EMT-MET switch in HNSCC cells. We showed that DDB2 repress EMT through increased expression of pro-EMT-TFs, *SNAIL* and *ZEB1*. These EMT-TFs interact with epigenetic regulators and target key epithelial genes [37]. Subsequently, we observed gain of epithelial marker E-cadherin and acute loss in mesenchymal markers, N-Cadherin and Vimentin, both at mRNA level and protein level in SCC9 cells (Figure 4). The ZEB1/ZEB2 and SNAIL superfamily are involved in direct repression of epithelial marker E-cadherin and increase in Vimentin [7]. However, we observed variations in expression of EMT-makers among cell types; inhibition of DDB2 did not induce an increase of mesenchymal marker Vimentin expression in SCC15:sh-DDB2 cells (not shown). It is possible that other mechanisms in those cells resist EMT-like changes. For example, knockdown of DDB2 was insufficient for an activation of *VEGF* expression in these cells (Figure 5B). In a previous study, we reported similar role of DDB2 in regulating EMT-TFs in colon adenocarcinoma cells [29]. Taken together, results presented in this study show that this prime function of EMT regulation by DDB2 is conserved among different tumor types.

To further explore the direct EMT-regulatory role of DDB2, we used DDB2 knockout (DDB2-KO) mice; we restored DDB2 expression in MEF-KO, the MEFs isolated from DDB2-KO mice. We showed that ectopic expression of DDB2 resulted in significant transcriptional repression of the same EMT-TFs *Snail* and *Zeb1*, and angiogenic factor *Vegf* in KO:DDB2 cells (Figure 5C). These results confirm the direct role of DDB2 in transcription regulation of the EMT-TF genes and show that the EMT-inhibitory role of DDB2 is conserved in mice.

Among different signaling pathways, VEGF induces angiogenesis and the TGFβs stimulate EMT [55]. TGFβs regulates a wide range of cellular functions including cell invasion and metastasis of cancer cells [56]. In this study, we observed that DDB2 preferentially represses *TGFB2* mRNA expression in HNSCC cells. Among different isoforms, TGFβ2 is over-expressed in glioblastoma multiforme (GBM), melanoma, colon cancer, breast and prostate cancer. In GBM, TGFβ2 has been shown to play essential role in pathogenesis [57]. In one study, the paracrine function of TGFb2 has been reported in cancer-associated fibroblasts (CAFs)-induced EMT of oral keratinocytes [41]. Identification of TGFB2 as a DDB2 target is a new significant observation. Our previous study shows that DDB2 recruits Suv39h methyl transferase on the promoters of *ZEB1* and *SNAIL* genes to increase H3K9-trimethylation and repression of these genes [29]. DDB2 also associates with EZH2 to regulatie expression of NEDD4L and RNF43 [39, 58]. Therefore, either an increase in the histone H3K27-trimethylation through EZH2 or an increase in H3K9-trimethylation through Suv39h could play a role in repression of *TGFB2* by DDB2. Further studies are required to analyze the mechanism of DDB2-mediated repression of *TGFB2* and to determine the role of *TGFB2* in EMT-progression in HNSCC.

EMT is a key regulator of metastasis, and in an orthotropic model of colon cancer in nu/nu mice, we showed that DDB2 blocks metastasis progression [29]. Monitoring DDB2 expression will be of therapeutic interest in the treatment of patients with advanced HNSCC. There are multiple stages to pharmacologically target EMT events in cancer. However, since there is a diversity of signals capable of inducing EMT within the tumor microenvironment, detailed analysis is required to determine whether increased DDB2 expression can prevent progression of HNSCC. Altogether, these observations suggest that lower DDB2 level in metastatic cancer acts as one of the major barriers of EMT and tumor progression. This study reveals an important tumor suppressor function of DDB2 in HNSCC.

## MATERIALS AND METHODS

### Cell culture

HNSCC cell lines, SCC4, SCC9, SCC15 and SCC25 were purchased from ATCC. Cells were cultured in 1:1 mixture of Dulbecco's modified eagle's medium and Ham's F12 with 10% FBS (Hyclone) supplemented with 200 uM L-glutamine, penicillin/streptomycin (100 Units), and 400 ng/ml hydrocortisone. Normal human oral keratinocytes (NHOK) cells immortalized with hTERT (HOK-TERT) were cultured in KBM Gold Keratinocyte growth medium from Lonza/Clonetics.

Plasmids, shRNA and Retroviral transduction: Human DDB2 cDNA with T7-tag was sub-cloned into the retroviral vector, pBABE-puro. Control-shRNA and DDB2-shRNAs were cloned into retroviral vector pSuper-retro-puro. The sequences for shRNAs were as follows: Control shRNA: *AGAACACGAGCACACACCA and* DDB2 shRNA: *GAGCGAGATCCGAGTTTAC*. The retroviruses were produced by transient transfection of the retroviral plasmid together with pIK packaging plasmid into tsa54 packaging cell line as described [59]. A pool of stable cells expressing T7-DDB2 or sh-DDB2 were generated by infection with the corresponding retrovirus followed by selection with puromycin.

### qRT-PCR analysis and Lists of primer sequences

Expression of T7-DDB2 and knockdown of DDB2 expression were confirmed by RNA analysis using qRT-PCR. Briefly, total RNA was isolated using Trizol (Zymo Research Corp). For RT-PCR, 2 μg of total RNA was reverse transcribed using M-MLV reverse transcriptase (USB Affymetrix) and Oligo dT primer. Authenticated PCR primers were purchased from Sigma and the qRT-PCR assays were performed using Ssoadvance SYBR kit on a CFX96 Real-Time PCR (Bio-Rad). Data were analyzed using Bio-Rad CFX Manager software package to determine Ct values and fold changes in expression by comparative Ct method. Primer sequences are included in the supplementary information (Supplementary Tables 1 and 2).

### Antibodies and western blot

Western bot assays were performed following previously described method (24). Cell lysates were made in RIPA buffer containing 20 mM Tris-HCL, 150 mM NaCl, 10% sodium deoxycholate, SDS (0.025%) supplemented with 1X protease inhibitor cocktail (Roche). The antibodies used in the study were DDB2 (#5416), N-Cadherin (#14215), Cell Signaling technology, E-cadherin (SC-7870), Vimentin (SC-6260), and HRP–conjugated secondary antibodies were purchased from Santa Cruz Biotechnology.

### Immunofluorescence

Immunofluorescence experiments were performed using cells grown on coverslips (24). Cells were incubated overnight with DDB2-Ab (Ab 77765, Abcam), Vimentin (SC-6260) and E-cadherin (SC-7870) from Santa Cruz Biotechnology at 1:200 dilution. The cells were washed five times with PBS followed by 1 h incubation at room temperature with anti-mouse secondary antibody Alexa Fluor 488 (Thermo Fisher Scientific) or anti-rabbit TRITC (Sigma). Coverslips were mounted onto glass slides using Vectashield Antifade mounting media with DAPI (Vector Laboratories). Images were acquired using a Zeiss LSM 510 microscope using 63x oil immersion objective. FITC conjugated Phalloidin was purchased from Sigma-Aldrich and used according to manufacturer's instructions.

### Tissue microarrays

Human normal and HNSCC tissues were obtained from US Biomax Inc. as paraffin embedded tissue microarray slides (HN811a; and HN242a). Tissues were deparaffinized and rehydrated in descending concentrations of ethanol as described before [40]. Endogenous peroxidase was quenched with 3% H2O2 for 15 min followed by blocking in normal serum for 1h. The tissues were incubated overnight at 4°C with the anti-DDB2 antibody (ab77765, Abcam) diluted (1:100) in 3% normal serum in a humidified chamber. The tissues were incubated first in biotinylated anti-rabbit antibody and then in ABC reagent using the Vectastain ABC kit PK-4001 as per manufacturer's instructions. The enzymatic reaction was detected by adding DAB substrate for 90 sec. After dehydration in ascending concentration of ethanol, the sections were mounted in permount (Thermo Scientific). The slides were imaged at 10x magnification for Normal tongue, Tumor Stages I–III/IV, and cancer adjacent normal tongue tissue (NAT) using a Zeiss Axio Observer D1 inverted microscope.

### Quantification/statistical analysis

Immunohistochemistry: Signal Intensities of at least five stained sections from each tumor tissue type (in triplicates) were quantified by ImageJ software as described in [60]. Intensity of DDB2 staining was estimated as average of five different areas per slide after background correction. Signal intensity of protein bands from Western blots were also quantified using ImageJ densitometry software. Statistical analyses were performed with GraphPad Prism software by using Student's unpaired *t*-test. A value of *P* < 0.05 was considered significant whereas a value of less than *P* < 0.01 was considered highly significant.

## SUPPLEMENTARY MATERIALS TABLES


