# Supplementary material for: DDB2 regulates Epithelial-to-Mesenchymal Transition (EMT) in Oral/Head and Neck Squamous Cell Carcinoma
Source: Oncotarget. 2018 Oct 5;9(78):34708–18. doi: 10.18632/oncotarget.26168 (PMC6205178; doi:10.18632/oncotarget.26168)
Supplement: Supplementary file 1 [file oncotarget-09-34708-s001.pdf]

DDB2 regulates Epithelial-to-Mesenchymal Transition (EMT) in Oral/Head and Neck Squamous Cell Carcinoma

SUPPLEMENTARY MATERIALS

Supplementary Table 1: List of primer sequences for human specific genes

Gene	Forward primer	Reverse primer
DDB2	CAAGGTTAGGGTTGGAGCAGG	AACTCTGGAGCAGTCTCTGGAGG
SNAIL	TTCAACTGCAAATACTGCAACAAG	CGTGTGGCTTCGGATGTG
ZEB1	GATGATGAATGCGAGTCAGATGC	ACAGCAGTGTCTTGTTGTTGT
VEGF	CTACCTCCACCATGCCAAGT	GCAGTAGCTGCGCTGATAGA
E-cad	GTCATCCAACGGGAATGCA	TGATCGGTTACCGTGATCAAAA
N-cad	TGCGGTACAGTGTAACTGGG	GAAACCGGGCTATCTGCTCG
VIM	TGCCGTTGAAGCTGCTAACTA	CCAGAGGGAGTGAATCCAGATTA
FIB	GGTGACACTTATGAGCGTCCTAAA	AACATGTAACCACCAGTCTCATGTG
TGFB1	CTAATGGTGGAAACCCACAACG	TATCGCCAGGAATTGTTGCTG
TGFB2	CCATCCCGCCCACTTTCTAC	AGCTCAATCCGTTGTTCAGGC
TGFB3	AACGGTGATGACCCACGTC	CCGACTCGGTGTTTTCCTGG
CYPA	GCAGACAAGGTCCCAAAGACAG	CACCCTGACACATAAACCCTGG
ACTB	AGGCGGACTATGACTTAGTTGCGTTACA	TGGCAAGGGACTTCCTGTAACAACGC

Supplementary Table 2: List of primer sequences for mouse specific genes

Gene	Forward primer	Reverse primer
Snail	GCCGGAAGCCCAACTATAGCGA	TTCAGAGCGCCCAGGCTGAGGTACT
Zeb1	GCTCCCTGTGCAGTTACACC	AGTGCACTTGAACTTGCGGT
Vegf	CTGTGCAGGCTGCTGTAACG	GTTCCCGAAACCCTGAGGAG
Tgfb1	GGAGAGCCCTGGATACCAAC	ATCCACTTCCAACCCAGGTC
Tgfb2	AGACAGTCCCAGGTGCTCTG	GACATCAAAGCGGACGATTC
Tgfb3	GAAGGCTGCACTCAGGAGAC	AGGTAATTCCTTTGGGGCAG
MnSod	ATTAACGCGCAGATCATGCA	TGTCCCCCACCATTGAACTT
18S	ACATCGACCTCACCAAGAGG	TCCCATCCTTCACATCCTTC
